# California provider and advocate perspectives about opportunities to optimize nutrition services and resources in the first 1000 days

**DOI:** 10.1002/rfc2.93

**Published:** 2024-06-11

**Authors:** Karen L. Lindsay, Trina Robertson, Helen Leka, Ashley Rosales, Jennifer T. Smilowitz, Candice Taylor Lucas

**Affiliations:** 1 Department of Pediatrics, UCI School of Medicine, University of California Irvine, Orange, California, USA; 2 UCI Susan Samueli Integrative Health Institute, Susan & Henry Samueli College of Health Sciences, University of California Irvine, Irvine, California, USA; 3 Dairy Council of California, Sacramento, California, USA; 4 UCI School of Medicine, University of California Irvine, Irvine, California, USA; 5 Department of Nutrition, University of California Davis, Davis, California, USA; 6 UCI Pediatric Exercise and Genomics Research Center, University of California, Irvine, Irvine, California, USA

**Keywords:** first 1000 days, food security, infant nutrition, nutrition equity, prenatal nutrition, public health nutrition

## Abstract

**Background:**

Nutrition in the first 1000 days of life, from conception to age 2 years, plays a critical role in shaping offspring’s physical and mental development, yet many families from underserved backgrounds suffer from nutrition inequity during this important stage of development. The objective of this study is to assess nutrition services and resources provided to families during the first 1000 days across diverse settings in California.

**Methods:**

A semistructured survey was disseminated to healthcare and educational providers who offer services to pregnant and/or postpartum women and children up to age 2 years. Perspectives about five domains of early-life nutrition services and resources were assessed: (1) accessibility, (2) mode of content delivery, (3) content of messages, (4) breastfeeding support, and (5) professional development on early-life nutrition. Mixed methodology was used to conduct descriptive and thematic analyses for closed and open-ended survey questions, respectively.

**Results:**

Survey respondents (*n* = 148) worked in healthcare (37%), governmental (20%), community (20%), and childcare settings (23%). Over 60% primarily served low-income families. Less than 9% reported that their organizations provide prenatal nutrition messaging about critical micronutrients required to support foetal neurodevelopment, highlighting an opportunity for professional development training in nutrition. Need for equitable access to nutrition education and resources by addressing Language, Income, Food resources, Time and Transportation (LIFTT) was a cross-cutting theme that emerged.

**Conclusion:**

Providers perceive a need to enhance LIFTT accessibility and improve delivery of early-life nutrition-related services for families in the first 1000 days by providing topic-specific education and culturally responsive resources with consistent, evidence-based messages.

## INTRODUCTION

Nutrition has far-reaching impacts on children’s overall health and well-being, affecting their ability to succeed in school and life.^1,2^ The first 1000 days of life, representing the period of earliest development from conception to age 2 years, is a critical window in which conditions, exposures, and behaviours may set the stage for later risk of neurodevelopmental conditions^3^ and chronic diseases, including obesity, diabetes, and hypertension.^4,5^ While there are multiple contributing factors, nutrition is a particularly salient one that exerts a strong influence from as early as conception.^6,7^ Maternal diet throughout pregnancy, infant feeding and weaning practices, and the quality and quantity of complementary foods and beverages each play an important role in early child growth and development. Further, healthy early life nutrition has been identified as a potentially protective factor against the deleterious health effects of adverse childhood experiences.^8,9^

The prevalence of childhood obesity and related chronic diseases is substantially higher among low-income and historically marginalized populations^10^ who often are reported to have greater risk of food insecurity, low health literacy, inadequate access to healthy food, low social support, and employment demands restricting breastfeeding practices, as well as time limitations on home food preparation.^11–13^ These health and nutrition inequities ultimately stem from structural racism, influenced by the historic footprints of redlining policies that limit access to supermarkets, healthy food, and medical care for under-represented minority groups.^14,15^ Maternal and child nutrition in the first 1000 days is disproportionately affected by these systemic structural inequities because of the significant increase in nutrient needs required to support optimal growth and development.^16,17^

Government agencies can influence the policies, systems, and environments that promote equitable access to nutritious food for families in the first 1000 days.^2^ In the United States, more than six million low-income mothers and children rely on Special Supplemental Nutrition Program for Women, Infants, and Children (WIC) for access to healthy, safe, and affordable foods and beverages, thus helping close nutrient gaps and improve nutrition security.^18^ CalFresh benefits, which is the Supplemental Nutrition Assistance Program (SNAP) in the state of California, additionally provides financial aid to low-income individuals to purchase certain restricted food and beverage items at grocery stores and farmer’s markets. Although these federal nutrition assistance programs provide a valuable safety net, additional supports are needed, as well as addressing structural inequities, to relieve the burden of food insecurity^19,20^ and provide nutrition security, defined as consistent access, availability, and affordability of foods and beverages that promote well-being and prevent disease.^21^ Health care and early care providers are vital frontline advocates facing the implications of systemic inequities in food access that manifest in physiologic health outcomes and impact growth and development.^22^ Yet, doctors and nurses who frequently interact with families during the first 1000 days have limited time and nutrition expertise to adequately address nutritional concerns,^23^ even for the most marginalized populations. Childcare providers frequently engage with families during this critical life stage and yet, delivery of healthy nutrition messaging to pregnant women and children up to age 2 is not typically incorporated into their services and providers may not feel adequately trained to educate on this topic.^24,25^

There is potential for a diverse array of trusted health care providers, educators and advocates to help close the gaps on the nutrition inequities experienced by pregnant and postpartum women and their young children from low income and historically marginalized populations. To this end, the first need is to assess the status of nutritional resources and services provided to this population. Thus, we conducted a mixed-methods survey among a sample of interdisciplinary leaders, healthcare providers and educators from a variety of nonprofit, governmental, childcare, and health care organizations in the state of California (collectively referred to as ‘service providers and advocates’ in this paper) that provide nutrition services and/or resources to families during the first 1000 days. The objective of this needs assessment survey was to gain insight and understanding of the barriers and challenges that these organizations face with respect to outreach to families, delivery of adequate nutrition education, and their ability to support access to nutritious food during this critical stage of early development.

## MATERIALS AND METHODS

### Study design and instrument development

A cross-sectional, web-based, semi-structured survey was created by eight multidisciplinary team members who were academic researchers, clinicians, and health educators with expertise in early life nutrition, maternal-child clinical practice and public health. Based on a combination of literature review and professional experiences, the group identified five key domains to be assessed by the survey as they specifically relate to the first 1000 days: accessibility of services, delivery of nutrition education, content of nutrition messages, breastfeeding support, and professional development opportunities on nutrition for providers and staff. Under the ‘content of nutrition messages’ domain, assessment of educational messaging that targets key nutrients of concern during the prenatal, infancy and toddlerhood stages was included (i.e., choline, iodine, iron, vitamin D), reflecting the nutritional needs highlighted in the 2020–2025 US Dietary Guidelines for Americans.^26^ We included the ‘professional development’ domain as it is frequently reported that certain service providers and advocates lack sufficient education on nutrition.^23^ Thus, we aimed to evaluate the current practices and interests in continuing education on nutrition that is specific to the first 1000 days to better support the needs of families in this life stage.

The multidisciplinary team initially proposed 30 survey questions spanning the five key domains described above. These were reviewed by the lead study investigators and an evaluation consultant, who was independent from the study team, to refine the content and reduce redundancy. Consensus was reached by the multidisciplinary team on 25 total survey questions, some of which were conditioned upon earlier responses within the survey (i.e., skip patterns). For example, if a respondent did not select ‘preconception’ or ‘pregnancy’ as life stages that were served by their organizations, subsequent questions related to the content of nutritional messages provided to pregnant clients were not presented to those respondents. The evaluation consultant assessed the structure and content of questions for cognitive validity and edited as required for improved readability.

Initial survey questions asked respondents about their professional role and characteristics of the organizations in which they work. Subsequent questions relating to the afore-mentioned domains either allowed multiple choice or free-text responses. The two questions that allowed free text responses were intended to gather input about respondents’ personal viewpoints on the barriers to accessing healthy nutrition among low-income families (Q6b), and suggestions for overcoming barriers to providing nutrition education and resources to families (Q15). Responses to these two questions were optional.

The final survey contents in English were translated to Spanish by a bilingual staff member and certified translator. The Spanish version was back-translated to English to ensure accuracy. English and Spanish versions of the survey were pilot tested by research personnel not involved in the survey development, and by community members representing our priority population, to detect and remove ambiguity and ensure appropriateness of length. The average length of completion time was 10 min. The full survey is available as Supporting Information.

### Procedures

The survey was built on the secure Research Electronic Data Capture (REDCap) web-platform and was distributed in November to December 2021. A public link to the online survey was disseminated to the target audience through email listservs, organizational newsletters, clinical departmental meetings, and colleagues of the study investigators. Further word-of-mouth dissemination was encouraged. Due to the open nature of survey dissemination, we were unable to determine response rate or characterize non-responders to the survey.

### Participants and recruitment

Purposeful sampling methods were utilized in recruitment prioritizing participation from service providers and advocates from any nonprofit, governmental, early childcare or health care organization in California that provides health and nutrition-related services or resources to families during the first 1000 days. Identifiable information was not collected from respondents, unless they voluntarily provided name and email address to enter a drawing for $50 gift cards as an incentive for survey completion. Due to the low-risk, anonymous nature of the study, the research was considered exempt from Institutional Review Board approval by University of California, Irvine.

### Data analysis

Survey data were downloaded from REDCap and imported to SPSS version 26.0 for analyses. Descriptive analyses were conducted to assess the frequency and mean values for responses to the multiple choice questions. Responses to select questions were displayed graphically using bar charts with stratification by respondents’ organization type to allow for visual comparison of responses across groups.

Two open-ended questions prompting free-text responses were analysed using iterative thematic analysis to identify and interpret patterns.^27^ The study team initially worked collaboratively to review and code free-text responses into thematic categories. On second review, thematic categories were explored separately by each author and consolidated into core themes for each survey question. On third review, all authors reconvened to discuss the selected core categories, resolve discrepancies by consensus, and decide on the final themes and subthemes. Significant quotes were identified from each of the two open-ended questions that best represented the themes and subthemes.

### Reflexivity

Our research team consisted of a highly educated group of academic researchers, clinicians, public health workers, and a medical student with expertise in food systems, nutrition policies, community nutrition, evaluation, health equity, maternal-child health, paediatrics, medical education, and culinary medicine. Among the six members, two identified as Non-Hispanic Black/African American (33.3%) and four as Non-Hispanic White (67.7%). Six members identified as female and four are mothers. Analysis and interpretation of the survey results was shaped by our educational, professional, and lived experiences. Throughout the interpretation of study findings, our research team met regularly to reflect on our identities and challenge any assumptions that emerged from the data.

## RESULTS

### Characteristics of respondents and their organizations

Unique survey responses were obtained from 148 individuals with representation across nine regions in the state of California (Supporting Information S1: Table 1). Response rates were more heavily weighted in regions where the target organizations had direct or indirect professional relationships with members of the study team. Respondents represented a diverse array of organization types including 37% (*n* = 55) from clinical/health care organizations, 20% (*n* = 30) from governmental agencies, 20% (*n* = 29) from community/nonprofit agencies, and 23% (*n* = 34) from childcare/preschool settings. Sixty-seven respondents identified as health care professionals spanning diverse disciplines: paediatrics (*n* = 22), dietetics (*n* = 18), nursing (*n* = 16), obstetrics/gynaecologist (*n* = 8), family medicine (*n* = 2), and paediatric dentistry (*n* = 1). Professions beyond health care included childcare/preschool providers, teachers, managers, and community health managers, while respondents from governmental agencies were primarily from county-level public health departments, eight of whom managed local WIC agencies. Individual demo-graphic characteristics such as sex, race, and age were not included in this study to support anonymity of study participants.

Table 1 describes the characteristics of the respondents’ organizations and services provided, stratified by organization type. More than one-third (34.5%) of respondents indicated that their organization serves >300 clients per month in the first 1000 days life stage. Proportions of MediCal eligible clients served ranged from 25% to 100%, with 88 respondents reporting 75%–100% were eligible. Word-of-mouth and provider referrals were the most frequently reported methods for clients within the first 1000 days hearing about services among the respondents’ organizations. About 82% of respondents, from both clinical and community settings, reported that their organizations provide referrals to WIC. However, only 46% of clinical and 69% of community-based respondents reported that their organizations refer clients to the CalFresh benefits program.

About two-thirds of respondents overall stated that their organizations provide some level of nutrition education to clients during the first 1000 days, although a substantially lower proportion of respondents from health care (60%) and childcare (44%) organizations reported providing these services. Healthcare and childcare organizations were also reported to have the lowest rate of provision of food resources or vouchers to clients (13% and 27%, respectively). Only 48% of all respondents reported that their organizations provided breastfeeding support to clients. These services were mostly provided by governmental agencies (67%).

### Accessibility of services

The majority of respondents reported the use of interpreters (72%) and/or translated written materials (80%) by their organizations to adapt their services to meet the linguistic and cultural needs of the communities they serve. However, only 32% reported translating social media posts to multiple languages, and 44% reported that messaging was tailored to meet cultural preferences.

An open-ended question asked respondents about their perception of additional needs for food resources among low-income families in the first 1000 days. Of the 39 respondents to this question (26% response rate), the thematic analysis of free-text responses revealed four key themes, as summarized in Supporting Information S1: Table 2, part A. As expected, ‘Knowledge and Skills’ acquired through enhanced nutrition education and practical skills training for providers was identified as a key theme. Another key theme identified was ‘Access’, that is, need for improved physical access to healthy food, financial means to purchase healthy foods, and access to federally funded and/or nonprofit community-based resources and services that provide food to families. ‘Navigation and Advocacy’ was a novel theme that emerged, and the quotes provided highlight the importance of having a trusted partner to connect and refer low-income families to the services and resources available within their community, while also helping them navigate the complicated systems that are often encountered with these services. The theme ‘Environment’ revealed basic needs within families’ home environments (i.e., space for food preparation and storage) that may represent barriers to achieving healthy nutrition regardless of level of food access, knowledge, and skills. The subtheme ‘Community Environment’, which includes housing and safety, was an additional structural factor highlighted that may impede families’ ability to procure healthy foods in certain areas.

### Delivery of nutrition education

**FIGURE 1:**
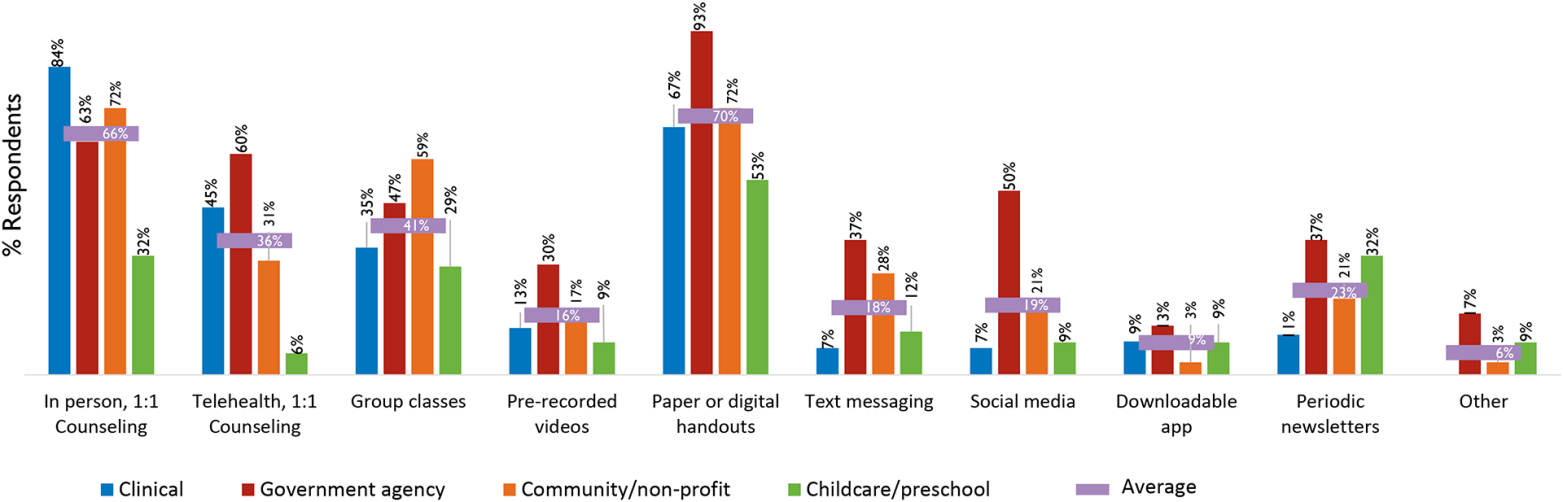
Modalities used to deliver nutrition education among respondents’ organizations, stratified by organization type (*n* = 147).

Figure 1 displays the modes of delivery for nutrition education to families in the first 1000 days, stratified by organization type. In-person 1:1 counselling and use of paper or digital handouts were the most frequently reported modes of delivering nutrition education. Respondents from governmental and clinical agencies also reported high utilization of telehealth counselling (60% and 45%, respectively) and, as expected, a substantially lower proportion of respondents from childcare agencies reported using this modality (6%). Prerecorded videos, text messaging, social media, and smartphone apps were less commonly (<20%) utilized modes of delivering nutrition education.

**FIGURE 2:**
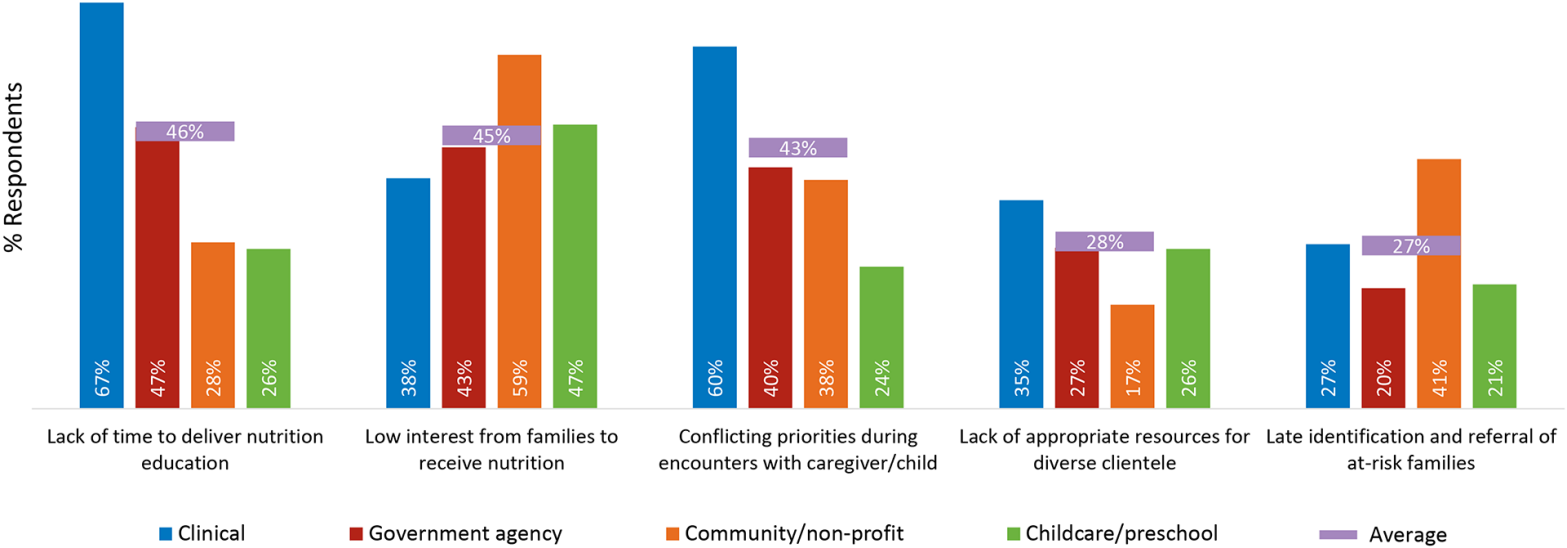
Challenges or barriers encountered by respondents’ organizations in providing nutrition education or resources during the first 1000 days (*n* = 148).

When asked about the challenges or barriers encountered by organizations in delivering nutrition education to families who are pregnant or with young children up to 2 years, lack of time and conflicting priorities during client encounters were the most cited barriers among clinical providers (Figure 2). A high proportion of respondents from community-based (59%) and childcare/preschool settings (47%) also cited low interest from families to receive nutrition education as a major barrier.

Fifty-nine respondents provided free-text responses to the open-ended question that asked how they believe such barriers to delivering nutrition education to families in the first 1000 days may be overcome (40% response rate). Thematic analysis of these responses identified five key themes, as outlined in Supporting Information S1: Table 2, part B. ‘Cultural Responsivity’ emerged as a prominent theme, which addressed not only the need for multilingual educational resources, but also educational content that is relevant and tailored to the unique needs and preferences of different cultural groups. The theme ‘Access’ also re-emerged in response to this question, which encompassed accessibility of educational content as well as accessibility of services within the complex network of medical and federal assistance programs. Under the theme ‘Structural Barriers’, respondents identified numerous action items required to help overcome structural inequities faced by low income and marginalized families.

### Content of nutrition education

Key nutrition messages delivered by respondents’ organizations were assessed according to life stage within the first 1000 days and the results are detailed in Supporting Information S1: Table 3. Among 81 respondents who reported providing services to pregnant women, we note that advice on prenatal supplementation was reported by 65% of respondents, but less than 9% reported providing education on food sources of key nutrients such as iodine and choline required to support optimal foetal development. Among 100 respondents who reported their organizations provide services to families with children around the time of transitioning to solids and other milk sources (6–12 months), guidance on supplementation of vitamin D or iron for babies was reported by 42% and 45% respectively, and only half of respondents reported that baby-led weaning was advised to clients.

### Breastfeeding support

Forty-eight percent of respondents (*n* = 74) reported providing services related to breastfeeding support. Of these, proper latching, reassuring families of the nutritional adequacy of breast milk, and access to lactation consultations were highlighted as the most important issues to address to ensure the adoption and continuation of breastfeeding. Nutritional needs and hydration of lactating mothers were the least commonly reported issues of concern in this regard. The most frequently cited factors considered to contribute to breastfeeding declines were the caregiver returning to work, lack of home support, and caregiver illness or depression. Among nine respondents who selected ‘other’ and subsequently provided a free-text response when asked about their perception of factors contributing to breastfeeding decline, five indicated that mothers’ lack of confidence or misunderstandings about breastfeeding and milk supply were key concerns.

### Professional development opportunities

**FIGURE 3:**
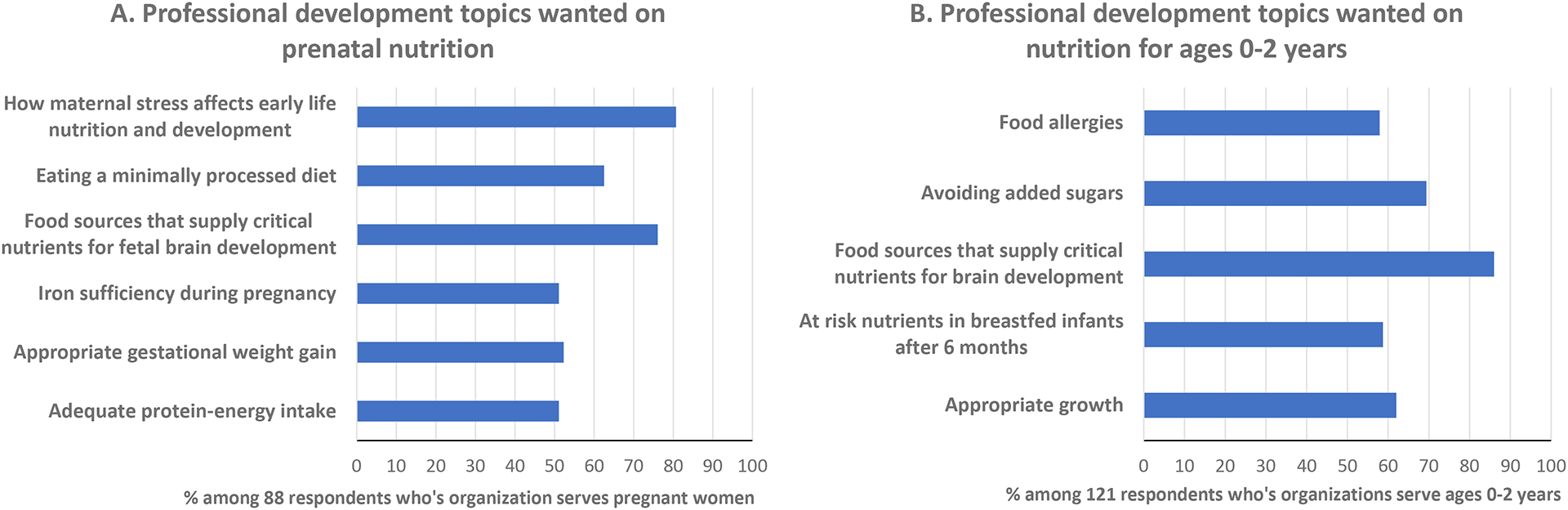
Interest in professional development topics on nutrition for (A) pregnancy and (B) children ages 0–2 years.

Twenty-six percent of respondents (including roughly one-third from clinical and childcare/preschool settings) reported that their organizations do not provide professional development opportunities on topics related to early life nutrition. Time, budgetary, and staffing constraints were the most frequently cited reasons for not receiving adequate professional development opportunities on this topic. As noted in Table 1, nearly 60% of respondents reported providing services to pregnant women. Among these respondents, interest for professional development training in nutrition topics was expressed, but particularly for prenatal nutrition to support foetal brain development (76%) and how maternal stress affects early life nutrition and development (81%) (Figure 3A). Among 121 respondents who provide services to families with children ages 0–2 years, interest for professional development training was expressed for topics in nutrition that support early child brain development (86%) and avoiding added sugars in the diet (69%) (Figure 3B).

## DISCUSSION

This mixed-methods, cross-sectional study identified several challenges and gaps in the content, provision, and delivery of nutrition-related services and resources to pregnant parents and families with children up to age 2 years residing in California. Accessibility and cultural responsiveness emerged as key themes in need of improvement at the organization and systems level. Gaps in the content of nutritional messaging were also identified, such that key micronutrients of concern during pregnancy, infancy and toddlerhood, and the optimal foods which provide these nutrients, were often not addressed. Diversity in modalities used to communicate nutritional messages to families appears to be lacking, particularly with respect to smartphone technology. Social and practical support for breastfeeding mothers is suboptimal, potentially contributing to early cessation of breastfeeding. Continued education on nutrition for early life development is needed and broadly desired, particularly among clinical providers and those in preschool/childcare settings, who may receive limited or no training in this field.

### Accessibility

Survey findings highlight that limited accessibility, on multiple levels, is a critical factor that may influence nutrition equity among families. This includes access to resources in appropriate Languages, as well as to Income, Food resources, Time and Transportation (LIFTT). The following sections discuss our survey findings through the lens of LIFTT accessibility.

#### LIFTT: Access to Linguistically and culturally relevant nutrition resources

Survey results suggest that inability to meet the unique linguistic needs and cultural experiences of some communities pose a barrier to providing families with relevant nutrition messages and educational resources in the first 1000 days. Although most respondents reported that translated written materials were made available by their organizations, few reported that social media postings were available in languages other than English. Furthermore, the contents of nutrition messaging may only be adapted to reflect cultural preferences less than half the time. This is an important consideration as diversity in traditional cuisines and culinary approaches to food preparation may make standard nutritional guidelines appear confusing or inappropriate.^28^ Developing more inclusive guidelines and educational resources that reflect the cultural differences and honour social practices of diverse populations are needed.^29^

#### LIFTT: Access to nutrition services with consideration for limited Income and Time

The need to improve accessibility in how nutrition education and support are delivered to families in the first 1000 days was also highlighted, with particular consideration for those with low income and time constraints. Caregiver work demands, caring for other children at home, and transportation barriers were some of the reasons mentioned that limit families from attending appointments or classes during which nutrition education and resources could be provided. While the growing popularity of telehealth may partially help to overcome these barriers,^30,31^ research has shown that families of low-income and low-educational status are least likely to engage in virtual or telephone health care appointments.^32^ Local community outreach programs that provide nutrition assistance through home visitation services on flexible schedules (e.g., lactation support, nutrition consultations for early child feeding) and greater leverage of online and social media platforms to disseminate on-demand information to families may help bridge these gaps.^33,34^ Furthermore, some families may be unaware of nutrition resources available in their community, or may feel confused or intimidated about how to enrol and obtain benefits from federal nutrition assistance programs.^35,36^ Our results indicate the need for greater promotion, navigation and advocacy within these systems to support low-income families’ access to nutrition resources by enhancing their sense of trust, safety and partnership. A recent systematic review suggests that interventions involving an online or virtual engagement component may be helpful for increasing WIC enrolment.^37^

#### LIFTT: Access to Food resources and Transportation

Despite large strides in public health efforts to provide much-needed access to food resources and nutrition counselling to low-income families throughout pregnancy and early childhood,^38^ results of this survey highlight inaccessibility to healthy food as an ongoing barrier for many families in California. Food deserts and food insecurity are two critical factors that adversely impact maternal and child health outcomes.^39^ Previous research has demonstrated that, even among families living within close proximity of retailers that accept WIC benefits, lack of transportation options among low-income families remains a persistent barrier to healthy food access.^40,41^ Results from the survey corroborate these findings as ‘lack of transportation’ was a frequently cited barrier to attaining healthy nutrition in the first 1000 days.

Through enhanced LIFTT accessibility, progress may be made towards achieving nutrition equity among families in the first 1000 days. However, issues beyond accessibility also exist, including the scope of nutrition related services and content of nutrition education provided to families, as well as the expertise of service providers and advocates in this space to provide nutrition-related education.

### Content of nutrition education

Survey results indicate that the content of nutritional messaging during the first 1000 days largely focuses on government-issued, evidence-based dietary guidelines for prenatal, infant, and early childhood nutrition. Few respondents reported providing specific counselling about key micronutrients of concern during this critical period of development and ways to obtain these specific nutrients through accessible foods, and culturally responsive diets. The 2020–2025 Dietary Guidelines for Americans recommends focusing attention on eating patterns for key life stages and nutrients of concern during the first 1000 days, specifically iodine and choline for pregnant and lactating women; iron for infants; and calcium, vitamin D, potassium, and dietary fibre for all age groups.^26^ However, a substantial proportion of pregnant and lactating women are reported to under consume micronutrients such as choline, iodine, vitamin C, copper, and magnesium, even taking into account intake from supplements.^42,43^ Others may be at risk of either under- or over-consuming folic acid and iron depending on usual dietary intake and supplement use.^42^ A recent nationwide study of WIC-enroled infants highlighted that less than 20% were meeting vitamin D recommendations during the first year of life.^44^ Thus, tailored dietary counselling may be required to support healthy pregnancy outcomes and optimal child growth and development. Importantly, people consume food, not nutrients in isolation, thus reinforcing the need for relatable, actionable nutrition education. For example, the simple inclusion of food such as eggs, milk, and cod in a person’s diet provides both iodine and choline,^45,46^ both of which are important nutrients during pregnancy and lactation.^47,48^

Several barriers in adequately delivering nutrition education to families were identified, including lack of time during encounters with medical doctors and a perceived low interest among families to receive nutrition education. However, previous qualitative research among caregivers of low-income children in California reported interest in receiving practical nutrition education.^49^ Some survey respondents highlighted the lack of access to dedicated dietitian nutritionists who are qualified to provide in-depth nutritional counselling to families during the prenatal, postpartum and early childhood life stages. A prior study that evaluated the perspectives of practitioners and parents about the impact of an enhanced paediatric community-based outpatient dietitian counselling services revealed several positive outcomes, including reduced hospital admissions and increased parental knowledge and confidence in healthy child feeding practices.^50^ Further implementation research is required in this area to justify the value of broad access to dietitian nutritionists for families in the first 1000 days.

### Breastfeeding support

Across the United States, breastfeeding rates dramatically decline across the first 6 months of life, such that only 56% of infants receive any breastmilk by age 6 months, only 25% are being exclusively breastfed, and these rates are even lower in infants from low-income communities.^51^ The suboptimal breastfeeding rates in the U.S. and especially within low-income communities are a result of a lack of federal paid family and medical leave that prevent women from having the time and support to initiate and sustain breastfeeding; a lack of flexibility and privacy for mothers to breastfeed or pump while at work; and difficultly affording lactation services, which are not part of standard care.^52–54^ Respondents to our survey who engage with lactating mothers highlighted the need for greater access to lactation consultations and social support services for families so that they may be empowered and adequately equipped to continue breastfeeding for longer periods. Unfortunately, postpartum lactation support provided by lactation consultants or lactation counsellors is not part of standard practice despite its efficacy in increasing breastfeeding initiation rates and improving breastfeeding rates.^55^ Respondents also acknowledged that guidance on pumping and storing breastmilk is required, which may help to address the top risk factor for breastfeeding decline, that is, caregivers returning to work. Comprehensive education about breast-feeding and lactation support is critical to provide to families early, beginning during pregnancy, as numerous respondents identified maternal lack of confidence in the first few weeks postpartum as a key reason for early cessation, which likely occurs even before caregivers return to work. Although not part of standard practice, prenatal lactation education increases breastfeeding intention, provides anticipatory guidance, and increases self-efficacy, which in turn, increases breastfeeding initiation and duration.^56^ Engagement in multisector collaboration is required by service providers and advocates to ensure consistent and accessible lactation and social support services are provided to families throughout pregnancy and postpartum, thus optimizing nutrition for the youngest members of our society.

### Opportunities for professional development on early-life nutrition

Continuing education opportunities focused on nutrition during the first 1000 days were reportedly lacking among one-third of survey respondents. There is a low emphasis on nutrition during medical school training, which likely persists throughout the career of medical doctors.^57^ However, survey respondents showed high interest in a variety of training topics related to nutrition during pregnancy and in early childhood. Making professional development training programs on optimal nutrition for the first 1000 days readily accessible for providers, such as through web-based platforms, may help to address these gaps.

### Strengths and limitations

This study is among the first to explore the knowledge, attitudes, and practices of service providers and advocates who address nutrition in the first 1000 days with California families. Study strengths include the broad inclusion of personnel across different organizational types, including organizations that serve a large proportion of low-income families. The combination of structured and unstructured survey questions facilitated a deeper understanding of the barriers and limitations that exist within the current systems that serve families in the first 1000 days. Although California represents a dense and diverse population within the United States, results of the survey may have limited generalizability nationally. Given the inherent limitations of community-level survey-based research, we were unable to determine the characteristics of nonresponders to the survey, nor were we able to capture perspectives and attitudes from all survey respondents for the optional open-ended questions that were analysed qualitatively. Thus, we cannot guarantee that the opinions of service providers across the entire state were adequately represented. However, responses to the open-ended questions were provided by 26% and 40% of total survey respondents respectively, indicating a moderate-high level of engagement among those who were surveyed. This survey did not ask respondents whether their organizations served families from primarily urban or rural settings, and it is possible that rural settings were underrepresented. Our survey was also limited by the fact it did not assess provider characteristics or explore potential influences of implicit bias or cultural humility in communication with families. Lastly, the survey did not assess the opinions of the end-users of interest, that is, the families. Further research is required to investigate experiences and attitudes of families toward nutrition and nutrition services during the first 1000 days so that comprehensive nutrition resources and programs tailored to their individual needs can be developed. Nevertheless, our study does provide vital information that can serve as a framework for further exploration of family attitudes and concordance or dissonance between family and provider perspectives about nutrition services during the first 1000 days of life.

### Implications for research and practice

The current systems of nutrition resource, support, and service provision supporting families in the first 1000 days in California are often fragmented across a range of organizations and implementation models. Without addressing structural competency to create coordinated and systemic changes to nutrition education and resource support,^58^ providing education and messaging that is inconsistent, or not culturally responsive, is likely to be ineffective.

**FIGURE 4:**
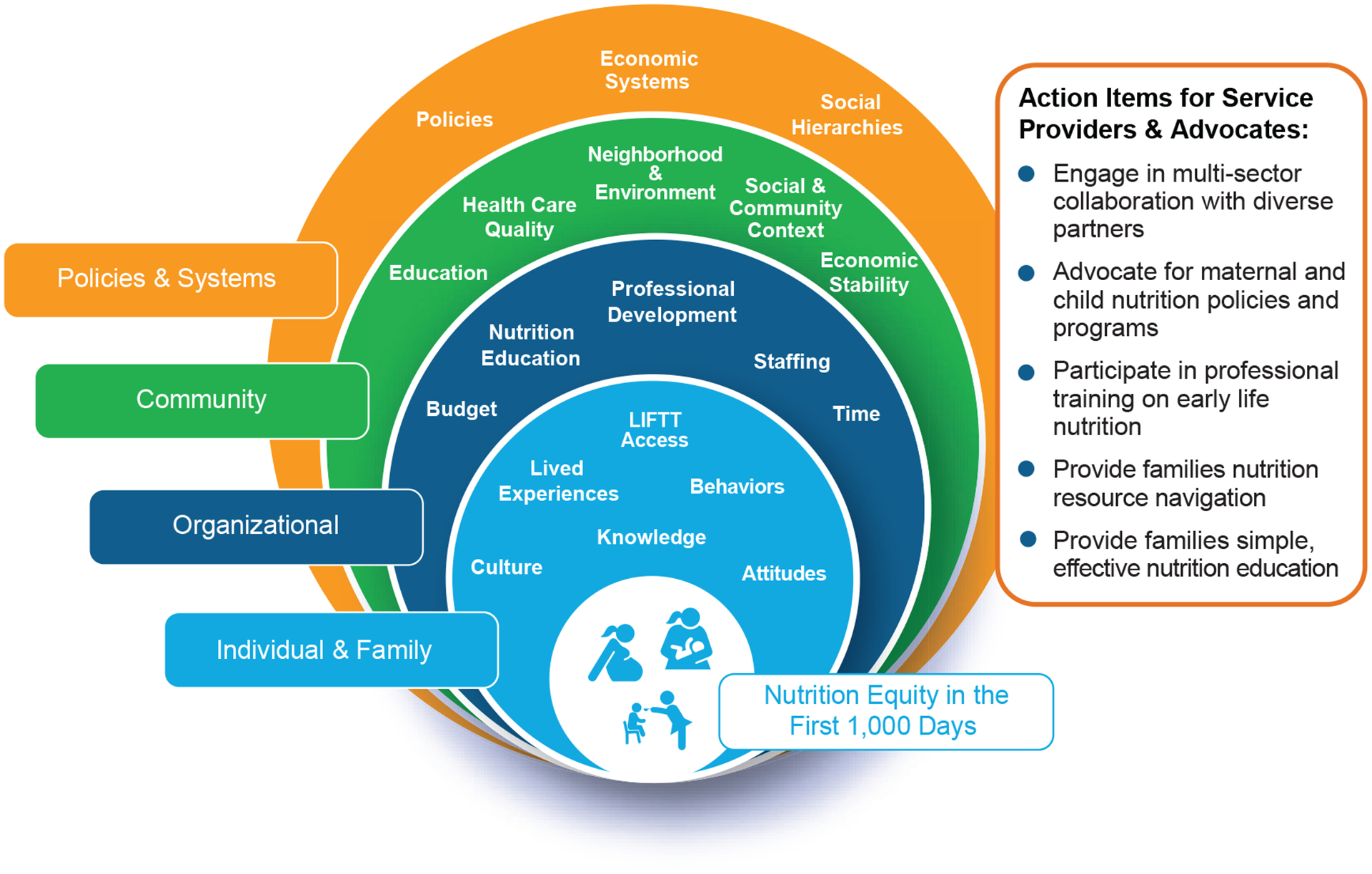
A socioecological approach for promoting nutrition equity in the first 1000 days. LIFTT, Language, Income, Food resources, Time, Transportation; these are key elements that impact accessibility to nutrition services and resources, as identified by the findings of our survey.

Figure 4 summarizes the authors’ perspectives about findings from this study through the lens of the socioecological model by highlighting key factors from the structural/macroenvironment level down to individual and family/interpersonal factors that influence nutrition equity among families in the first 1000 days. The results of this study highlight opportunities for improvement in the content and delivery of nutrition services and resources to families. Thus, in Figure 4 we propose five action items that are required to promote nutrition equity in the first 1000 days. While these action items are primarily geared towards service providers and advocates who serve families during these life stages, we acknowledge the need for multisector collaboration and community-driven solutions for achieving nutrition security.

Comprehensive educational programs are needed that include practical skills-based learning to enhance knowledge around food procurement, preparation, and storage in a culturally responsive way, and awareness of resources that directly provide high-quality, nutritious foods to low-income families. There is also room to expand the use of technology to provide both passive and interactive nutritional support that is available on demand. Furthermore, increased access to dietitian and lactation consultations during the first 1000 days may improve families’ food choices, ensure nutrient needs are met, and support initiation and continuation of breastfeeding. Early intervention is recognized as an ideal approach to supporting long-term health, so directing resources and efforts to increase access to nutritious foods, such as utilization of federal nutrition assistance programs, and nutrition education for the preconception life stage, including younger women of reproductive age who are not yet contemplating pregnancy, may yield improved health outcomes for future generations.^7^ Finally, addressing the social and structural determinants of health, and systemic barriers impacting communities at increased risk of cardiometabolic health issues, is critical.

## CONCLUSION

In conclusion, this survey of California service providers and advocates who interact with families in the first 1000 days revealed several barriers to achieving nutrition equity for families, including poor accessibility of services, incomplete or suboptimal nutrition information, inadequate breastfeeding support services, and insufficient opportunities for professional development on nutrition for early life development. However, opportunities for improvement across these areas were also identified and we propose action items to improve the competency and content of nutrition resources and services that are provided to families in the first 1000 days. Achieving nutrition security through enhanced LIFTT access and culturally responsive service provision during the first 1000 days will ultimately require multisector collaboration, advocacy, and action to fully support families where they live, learn, work, play, and gather.

## Supplementary Material

Suppl Tables

Suppl Material_survey

## Figures and Tables

**TABLE 1:** Characteristics of respondents’ organizations and the services they provide, stratified by organization type.

	Total (*n* = 148)	Health care (*n* = 55)	Governmental agency (*n* = 30)	Community/nonprofit (*n* = 29)	Childcare/preschool (*n* = 34)
Life stages of clients served					
Pre-conception	60 (40.5)	33 (60.0)	17 (56.7)	8 (27.6)	2 (5.9)
Prenatal	88 (59.5)	34 (61.8)	26 (86.7)	22 (75.9)	6 (17.6)
Postnatal	91 (61.5)	37 (67.3)	26 (86.7)	22 (75.9)	6 (17.6)
Infancy (0–12 months)	116 (78.4)	41 (74.5)	29 (96.7)	22 (75.9)	24 (70.6)
Ages 1–2 years	121 (81.8)	42 (76.4)	29 (96.7)	19 (65.5)	31 (91.2)
Number of clients served per month					
<100	71 (48.0)	18 (32.7)	9 (30.0)	14 (48.3)	30 (88.2)
100–300	24 (16.2)	13 (23.6)	6 (20.0)	3 (10.3)	2 (5.9)
300+	51 (34.5)	23 (41.8)	15 (30.0)	11 (37.9)	2 (5.9)
Proportion of clients who are Medical eligible					
About 25%	11 (7.4)	7 (12.7)	0 (0.0)	2 (6.9)	2 (5.9)
About 50%	18 (12.2)	10 (18.2)	4 (13.3)	2 (6.9)	2 (5.9)
About 75%	61 (41.2)	19 (34.5)	15 (50.0)	14 (48.3)	13 (38.2)
100%	27 (18.2)	10 (18.2)	7 (23.3)	5 (17.2)	5 (14.7)
Unknown	27 (18.2)	7 (12.7)	3 (10.0)	6 (20.7)	11 (32.4)
None	3 (2.0)	2 (3.6)	0 (0.0)	0 (0.0)	1 (2.9)
How clients are referred to the organization					
Brochures	79 (53.4)	22 (40.0)	21 (70.0)	24 (82.8)	12 (35.3)
Word of mouth	128 (86.5)	44 (80.0)	28 (93.3)	27 (93.1)	29 (85.3)
Social media	80 (54.1)	26 (47.3)	18 (60.0)	20 (69.0)	16 (47.1)
Website	94 (63.5)	35 (63.6)	21 (70.0)	23 (79.3)	15 (44.1)
Referred by other providers	115 (77.7)	42 (76.4)	26 (86.7)	25 (86.2)	22 (64.7)
Targeted outreach	68 (45.9)	14 (25.5)	23 (76.7)	21 (72.4)	10 (29.4)
Other	10 (6.8)	4 (7.3)	3 (10.0)	0 (0.0)	3 (8.8)
Types of services provided					
Physical exams/well checks	48 (32.4)	39 (70.9)	3 (10.0)	4 (13.8)	2 (5.9)
Food resources or vouchers	49 (33.1)	7 (12.7)	17 (56.7)	16 (55.2)	9 (26.5)
Breastfeeding/lactation support	71 (48.0)	28 (50.9)	20 (66.7)	15 (51.7)	8 (23.5)
Parenting classes	48 (32.4)	14 (25.5)	7 (23.3)	16 (55.2)	11 (32.4)
Mental health services	54 (36.5)	23 (41.8)	7 (23.3)	16 (55.2)	8 (23.5)
Nutrition education	96 (64.9)	33 (60.0)	26 (86.7)	22 (75.9)	15 (44.1)
Childcare	39 (26.4)	0 (0.0)	2 (6.7)	7 (24.1)	30 (88.2)
Caregiver-child play groups	16 (10.8)	0 (0.0)	4 (13.3)	6 (20.7)	6 (17.6)
Resources and referrals	99 (66.9)	38 (69.1)	26 (86.7)	21 (72.4)	14 (41.2)
Other	18 (12.2)	8 (14.5)	6 (20.0)	2 (6.9)	2 (5.9)

*Note*: All data are displayed as *n* (%). For some characteristics, the sum of percent values within categories exceed 100 where multiple responses were permitted.

## Data Availability

The data that support the findings of this study are available from the corresponding author upon reasonable request.
