# Supplementary material for: California provider and advocate perspectives about opportunities to optimize nutrition services and resources in the first 1000 days
Source: Reprod Female Child Health. Author manuscript; Available in PMC 2025 Jan 17. (PMC11741669; doi:10.1002/rfc2.93)
Supplement: Suppl Tables [file NIHMS2007619-supplement-Suppl_Tables.docx]

**Supplemental Table 1: Number of survey respondents according to California region of service (n=148)**

| **California Region** | **n (%)** |
| --- | --- |
| Central Coast | 20 (13.5) |
| Central Valley | 35 (23.6) |
| Far North | 1 (0.7) |
| Inland Empire | 3 (2.0) |
| Los Angeles County | 15 (10.1) |
| Orange County | 39 (26.4) |
| Sacramento Region | 22 (14.9) |
| San Diego / Imperial Valley | 7 (4.7) |
| San Francisco / Bay Area | 5 (3.4) |
| Statewide | 1 (0.7) |

**Supplemental Table 2:** **Key themes, subthemes and supporting quotes from qualitative survey questions**

| **Related themes and subthemes** | **Illustrative Quote(s)** |
| --- | --- |
| ***A. What additional needs do you think families have when considering food resources?*** | |
| *Theme 1: Knowledge and Skills* |  |
| - Nutrition education | “How to prepare healthy meals within a budget and using seasonal vegetables and fruits” |
| - Food utilization and management | “Information on how to cook in a healthful way. Education on how to select and store fresh fruits and vegetables and other whole foods.”  “Food Waste Management skills” |
| - Behavior management | “Seems we give them the healthy foods so they spend their money on unhealthy fast foods to reward their child – to make them happy” |
| - Point of purchase decisions | “I would say guidance in deciding what foods should be prioritized in their search for nutritious foods” |
| *Theme 2: Navigation and Advocacy* |  |
| - Awareness and connection | “They are unaware of the resources that are available to them. There is a need for organizations to educate the parents on what resources are available for them and help them access them.” |
| - Support | “...The back and forth between Medi-Cal managed care/providers/WIC office adds undue stress on the client” |
| *Theme 3: Access* |  |
| - Access to healthy food | “They probably would benefit from access to better food. Due to food bank providing almost expired food. They deserve fresh food for their families! Or vouchers once in a while to certain stores for meat purchases or items that are harder to get.”  “More access to fruits and vegetables that are organic.”  “They need more access to different choices of food.”  “Time to prepare food” |
| - Access to food resources | “Transportation issues to receive food resources”  “Delivery of food since some must use public transportation.”  “Lack of transportation to get to food banks.”  “Food vouchers and food bank”  “...$ to buy food to supplement what they get free”  “low cost or no cost community food resources” |
| *Theme 4: Environment* |  |
| - Home environment | “...space to prepare and store food”  “Not enough proper storage in their homes to preserve these foods.” |
| - Community environment | “We are in a rural area and the cost of gas has become an increasing hardship when trying to access food resources.”  “Walkable, in neighborhood access to appropriate food.”  “Housing and safe environments” |
| ***B. What suggestions do you have for helping families overcome barriers to receiving nutrition education?*** | |
| *Theme 1: Timing & Delivery of Nutrition Education* |  |
| - Intervene early in the preconception/prenatal life stages | “Providing education during prenatal. Parents are exhausted and face challenges after baby is born and are more likely to remember or utilize the information before baby is born”  “Targeting moms at early conception and then at postpartum check” |
| - Provide cooking and food preparation skills training | “I think families would benefit from classes or at home teaching. When I was a kid someone from public health came to our home to teach my mom how to can food and make meal plans”  “How to make quick meals that are not the typical sandwich/wrap/salad/soups that we see in all the healthy eating swap brochures” |
| - Utilize a variety of educational modalities | “Providing as many options to receive the education.”  “Send out video resources regarding nutrition!” |
| - Acknowledge competing challenges for time | “For many families struggling to meet daily demands of life, nutrition education may not be a top priority” |
| *Theme 2: Cultural Responsivity* |  |
| - Provide multilingual resources | “Meet the cultural and linguistic needs of the family”  “Information available in more languages. Local school districts have 46 languages represented; most written materials are available in 3 or 4 languages max.”  “Some people in our community have difficulty reading or understanding Spanish since they are indigenous and speak a dialect.” |
| - Acknowledge cultural traditions and beliefs | “Provide more culturally relevant nutritional meal planning with demonstration/outreach”  “Make some cultural foods like mole, rice dishes, etc.” |
| - Tailor resources to families’ preferences and needs | “Asking families for their input on what they will like to be informed in and what times and days they are available to meet for classes or to pick up resources.”  “Needs to be relevant for the participant.” |
| - Empower and motivate caregivers | “For parents to speak up when they need the help on low on resources”  “I used to hold monthly “Coffee Klatches” for Spanish-speaking families...they’d ask questions, and I’d provide resources for many basic needs such as food, clothing, housing, and medical care. I’d also accompany new moms to the WIC office to provide transportation and moral support for the education before receiving the milk and cereal. I often interpreted, but also, just offered care and support.” |
| *Theme 3: Access* |  |
| - Diversify modes of delivery | “Most clients/families have access to smart phones or computers so having downloadable links and or apps could help our clients access education without using up so much paper. I think most handouts end up getting lost or thrown in the trash at some point.” |
| - Utilize plain language, simple messaging | “Providing a simple, easy to understand guide on the best most cost effective way to provide healthy nutrition to children.” |
| - Optimize efficient systems and navigation | “Access right away when families are in need. Placing a referral and waiting for someone to get back or follow-through is not good. Then we don't always get a response from the agencies and we lose the family because they didn't get services.”  “Have a diagram of what to expect at medical visits with the respective timeline and embed this in the normal cycle of care for the provider, the patient-family, and the front desk.”  “Connecting families with WIC, SNAP” |
| *Theme 4: Structural Barriers* |  |
| - Provide a safe and trusted environment | “Provide safe places for this education. Safe can mean a place where families feel they will not be bullied or if they are immigrants that they will not be turned in to immigration police.”  “If not legal citizens we need to reassure them we don't share demographics with authorities to receive our services.”  “It is sometimes scary to go to a government agency for help, particularly when you do not speak the language and fear bullying, hate crimes, and shaming” |
| - Support low-income families | “Grants to support nutrition education during this time period for Non-MediCal patients that have a financial need”  “Getting local farmers markets going for healthy options even in urban areas.” |
| - Address transportation challenges | “Make it accessible over the phone rather than requiring in person, to improve efficiency and remove transportation barriers.”  “Have list of grocery stores that accept EBT/SNAP in their area/walking distance” |
| - Improve access to clinical nutrition services | “Expanding appointment times to allow time for the discussion. Being able to bill for follow-up nutritional counseling appointments.” “Need some person and funding dedicated to nutrition counseling, resources for affordable options.” |
| *Theme 5: Professional Development* |  |
| - Target diverse educators/advocates | “Our course could be adapted for any setting, not just a college course for credit.” |
| - Promote community engagement | “I teach nursing students for their community health/population health courses. I would love to get my students more involved in nutrition education through partnering with an agency/office.”  “Through food program maybe provide training for providers” |

**Supplemental Table 3: Key nutritional messages provided by respondents’ organizations at each life stage during the first 1000 days, and top nutrition concerns heard from families**

|  | **Total** | **Healthcare** | **Governmental agency** | **Community/ non-profit** | **Childcare/ preschool** |
| --- | --- | --- | --- | --- | --- |
| **Mothers’ nutrition during pregnancy [N respondents]** | 81 | 33 | 23 | 19 | 6 |
| General healthy eating with MyPlate and food groups | 69 (85.2) | 25 (75.8) | 22 (95.7) | 18 (94.7) | 4 (66.7) |
| Staying within gestational weight gain recommendations | 57 (70.4) | 24 (72.7) | 17 (73.9) | 14 (73.7) | 2 (33.3) |
| Nutrition for gestational diabetes or other pregnancy complications | 60 (74.1) | 25 (75.8) | 17 (73.9) | 15 (78.9) | 3 (50.0) |
| Prenatal supplementation | 53 (65.4) | 23 (69.7) | 14 (60.9) | 13 (68.4) | 3 (50.0) |
| Food sources of choline | 7 (8.6) | 1 (3.0) | 2 (8.7) | 4 (21.1) | 0 |
| Food sources of iodine | 7 (8.6) | 1 (3.0) | 2 (8.7) | 4 (21.1) | 0 |
| Food sources of iron | 44 (54.3) | 15 (45.5) | 18 (78.3) | 11 (57.9) | 0 |
| **Mothers’ nutrition during breastfeeding [N respondents]** | 74 | 27 | 22 | 20 | 5 |
| General healthy eating with MyPlate and food groups | 61 (82.4) | 18 (66.7) | 21 (95.5) | 19 (95.0) | 3 (60.0) |
| Staying within weight loss recommendations | 33 (44.6) | 12 (44.4) | 9 (40.9) | 11 (55.0) | 1 (20.0) |
| Staying hydrated | 63 (85.1) | 22 (81.5) | 18 (81.8) | 18 (90.0) | 5 (100.0) |
| **Infant feeding [N respondents]** | 96 | 35 | 24 | 17 | 20 |
| Identifying hunger and satiety cues | 83 (86.5) | 31 (88.6) | 24 (100.0) | 16 (94.1) | 12 (60.0) |
| Supporting breastfeeding continuation | 90 (93.8) | 35 (100.0) | 24 (100.0) | 15 (88.2) | 16 (80.0) |
| Bottle feeding | 64 (66.7) | 22 (62.9) | 17 (70.8) | 11 (64.7) | 14 (70.0) |
| Water and beverage needs | 61 (63.5) | 22 (62.9) | 13 (54.2) | 14(82.4) | 12 (60.0) |
| Vitamin D supplementation | 48 (50.0) | 25 (71.4) | 14 (58.3) | 7 (41.2) | 2 (10.0) |
| Iron supplementation | 44 (45.8) | 22 (62.9) | 11 (45.8) | 8 (47.1) | 3 (15.0) |
| **Children transitioning to solid foods and other milk sources [N respondents]** | 100 | 37 | 24 | 16 | 23 |
| Transitioning from breastmilk/formula to dairy or soy milk | 71 (71.0) | 27 (73.0) | 21 (87.5) | 9 (56.3) | 14 (60.9) |
| Transition from bottle to cup | 79 (79.0) | 26 (70.3) | 22 (91.7) | 12 (75.0) | 19 (82.6) |
| Introducing new foods | 89 (89.0) | 34 (91.9) | 21 (87.5) | 13 (81.3) | 21 (91.3) |
| Baby led weaning | 51 (51.0) | 21 (56.8) | 12 (50.0) | 11 (68.8) | 7 (30.4) |
| Exposure to potential allergenic foods | 63 (63.0) | 26 (70.3) | 15 (62.5) | 9 (56.3) | 13 (56.5) |
| Adequate water intake | 70 (70.0) | 24 (64.9) | 18 (75.0) | 11 (68.8) | 17 (73.9) |
| Avoiding non-recommended beverages (e.g., sodas, milk alternatives) | 81 (81.0) | 28 (75.7) | 20 (83.3) | 13 (81.3) | 20 (87.0) |
| Vitamin D supplementation | 42 (42.0) | 22 (59.5) | 9 (37.5) | 7 (43.8) | 4 (17.4) |
| Iron supplementation | 45 (45.0) | 23 (62.2) | 10 (41.7) | 8 (50.0) | 4 (17.4) |
| **Feeding children ages 1 to 2 years [N respondents]** | 102 | 34 | 26 | 15 | 27 |
| Parental feeding styles | 51 (50.0) | 17 (50.0) | 17 (65.4) | 6 (40.0) | 11 (40.7) |
| Avoiding non-recommended beverages (e.g., sodas, milk alternatives) | 81 (79.4) | 28 (82.4) | 23 (88.5) | 13 (86.7) | 17 (63.0) |
| Juice consumption | 78 (76.5) | 26 (76.5) | 22 (84.6) | 9 (60.0) | 17 (63.0) |
| Picky eating | 81 (79.4) | 27 (79.4) | 23 (88.5) | 9 (60.0) | 22 (81.5) |
| Adequate iron intake to reduce risk of anemia | 54 (52.9) | 25 (73.5) | 12 (46.2) | 9 (60.0) | 8 (29.6) |
| Healthy snacks | 93 (91.2) | 30 (88.2) | 24 (92.3) | 13 (86.7) | 26 (96.3) |
| Water intake | 76 (74.5) | 25 (73.5) | 19 (73.1) | 11 (73.3) | 21 (77.8) |
| Avoiding added sugars | 83 (81.4) | 30 (88.2) | 24 (92.3) | 11 (73.3) | 18 (66.7) |

Data presented as N(%).
