# Supplementary material for: California provider and advocate perspectives about opportunities to optimize nutrition services and resources in the first 1000 days
Source: Reprod Female Child Health. Author manuscript; Available in PMC 2025 Jan 17. (PMC11741669; doi:10.1002/rfc2.93)
Supplement: Suppl Material_survey [file NIHMS2007619-supplement-Suppl_Material_survey.pdf]

First 1000 Days Nutrition Needs Assessment

Thank you sincerely for agreeing to participate in this survey. We want to hear about your perspective on providing services for early life nutrition through your organization.

This brief survey will take approximately 10 minutes to complete and all of your responses will remain confidential. Those who complete the survey and provide contact information (at the end of the survey) will be eligible to win one of four $50 gift cards.

[redacted]

Thank you!

Please respond to every question to the best of your ability.

1. At what type of organization do you work?

- ◯ Community-based agency/non-profit
- ◯ Government agency (e.g., WIC)
- ◯ School/Preschool
- ◯ Clinic, hospital, or other health setting
- ◯ Child-care center or setting
- ◯ Academic setting (e.g., university)
- ◯ Food bank
- ◯ Other

Other (please specify):

2. What is your role at your organization?

- ◯ Case manager
- ◯ Nurse/Nurse Practitioner
- ◯ Registered Dietitian Nutritionist
- ◯ Medical Doctor
- ◯ Health Educator
- ◯ Community Health Manager (or Community Based Leader)
- ◯ Food Service Professional
- ◯ Other

Other (please specify):

2a. What is your area of medical specialty?

- ◯ Obstetrics/Gynecology
- ◯ Pediatrics
- ◯ Family Medicine
- ◯ Other

Other (please specify):

3. We are interested in finding out more about the first 1000 days of life and families' nutritional needs during the timeframe. The term "first 1000 days" refers to the period from conception until child is age 2 years, which is a period of rapid growth and development.

What is your impression of the term "first 1000 days"?

- ◯ I understand and like the term "first 1000 days"
- ◯ I find the term "first 1000 days" unclear
- ◯ I prefer the term "first 1000 days of life"
- ◯ I prefer the term "early years of life"
- ◯ I prefer the term "early life development"
- ◯ Other suggestions/comments

Other suggestions/comments:

4. For which life stage(s) does your organization provide resources and/or services for? Select all that apply.

- ☐ Preconception
- ☐ Pregnancy
- ☐ Postnatal
- ☐ Infancy
- ☐ Ages one to two

5. Which type of participants access your organization's services? Select all that apply.

- ☐ Mother
- ☐ Child
- ☐ Father
- ☐ Other caregiver (e.g. grandparents, nanny, foster parent)
- ☐ Other

Other (please specify):

When referring to participants in future questions, we will use the term "families" to include mother, father, child, and other caregivers to whom services and resources are delivered to during the first 1000 days.

6. What type of services do you provide? Select all that apply.

- ☐ Physical exams/well checks
- ☐ Food resources or vouchers
- ☐ Breastfeeding/Lactation support
- ☐ Parenting classes
- ☐ Mental health services or support
- ☐ Nutrition education
- ☐ Childcare
- ☐ Caregiver-child play groups (i.e., Mommy & Me classes)
- ☐ Resources and referrals
- ☐ Other

Other (please specify):

6a. What type of food resources do you provide?

- ☐ Serve prepared foods (i.e., daycare meals)
- ☐ Provide food vouchers
- ☐ Provide unprepared foods (food bank)
- ☐ Other

Other (please specify):

6b. What additional needs do you perceive low-income families have for food resources?

7. To which of the following agencies does your organization provide referrals? Select all that apply.

- ☐ WIC
- ☐ SNAP/CalFresh
- ☐ Food banks/food pantries
- ☐ Other
- ☐ None

Other (please specify):

8. Approximately how many clients within the first 1000 days life stage (conception to age two years) does your organization serve per month?	◯ < 20<br>◯ 20-50<br>◯ 51-100<br>◯ 100-150<br>◯ 150-200<br>◯ 200-300<br>◯ 300-400<br>◯ 400+
9. What proportion of your organization's clients are on Medical or would be Medical eligible?	◯ All<br>◯ About 75%<br>◯ Half<br>◯ About 25%<br>◯ None<br>◯ Unknown
10. What are some of the ways clients are referred to or hear about your organization's services? Select all that apply.	☐ Brochures<br>☐ Word of mouth<br>☐ Social media<br>☐ Website<br>☐ Referred by other providers<br>☐ Targeted recruitment or outreach<br>☐ Other
Other (please specify):	
11. What are some of the ways your organization tailors its services to meet the linguistic and cultural needs of the communities you serve? Select all that apply.	☐ Interpreters<br>☐ Peer educators<br>☐ Translated written materials<br>☐ Social media posts in multiple languages<br>☐ Tailored content to cultural preferences<br>☐ Other
Other (please specify:)	
12. When during the first 1,000 days do you consider families most open to learning about nutrition, if at all?	☐ Preconception<br>☐ Early pregnancy (weeks 1-20)<br>☐ Late pregnancy (weeks 21-40)<br>☐ During breastfeeding<br>☐ Transition to daycare<br>☐ Weaning to solid foods<br>☐ Other
Other (please specify):	

13. Which of the following modalities, if any, does your organization use for nutrition education? Select all that apply.	☐ In person, one on one counseling<br>☐ Telehealth, one on one counseling<br>☐ Group class<br>☐ Pre-recorded videos<br>☐ Paper or digital handouts<br>☐ Text messaging<br>☐ Social media<br>☐ Downloadable app<br>☐ Periodic newsletters<br>☐ Other
Other (please specify):	
14. What are some of the challenges or barriers your organization encounters in providing nutrition education or resources during the first 1,000 days to the families you serve? Select all that apply.	☐ Lack of time to deliver nutrition education<br>☐ Low interest from families to receive nutrition education/resources<br>☐ Conflicting priorities during encounters with caregiver/child (e.g. medical conditions)<br>☐ Lack of appropriate resources for diverse clientele<br>☐ Late identification and referral of at-risk families<br>☐ Other
Other (please specify):	
15. Please provide any suggestions you may have for helping families overcome the barriers or challenges they may face in receiving nutrition education.	
16. What life stage(s) does your organization focus nutrition education during the first 1,000 days? Select all that apply.	☐ Mother's food intake during pregnancy<br>☐ Mother's food intake during breastfeeding<br>☐ Infant feeding<br>☐ Child transitioning to solid foods and other milk sources<br>☐ Feeding 1 to 2 years old child<br>☐ N/A
16a. What are the key nutritional messages your organization provides regarding mothers' food intake during pregnancy? Select all that apply.	☐ General eating with MyPlate and food groups<br>☐ Staying within weight gain recommendations for pregnancy<br>☐ Nutrition for gestational diabetes or other pregnancy complications<br>☐ Prenatal supplementation<br>☐ Food sources of choline<br>☐ Food sources of iodine<br>☐ Food sources of iron<br>☐ Other
Other (please specify):	
16b. What are the key nutritional messages your organization provides regarding mothers' food intake during breastfeeding? Select all that apply.	☐ General eating with MyPlate and food groups<br>☐ Staying within weight loss recommendations<br>☐ Staying hydrated<br>☐ Other
Other (please specify):	

16c. What are the key nutritional messages your organization provides regarding infant feeding? Select all that apply.	☐ Identifying hunger and satiety cues<br>☐ Supporting breastfeeding continuation<br>☐ Bottle feeding<br>☐ Water and beverage needs<br>☐ Vitamin D supplementation<br>☐ Iron supplementation<br>☐ Other
Other (please specify):	
16d. What are the key nutritional messages your organization provides regarding children transitioning to solid foods and other milk sources? Select all that apply.	☐ Transitioning from breast/formula to dairy milk or soy<br>☐ Transition from bottle to cup<br>☐ Introducing new foods<br>☐ Baby led weaning<br>☐ Exposure to potential allergenic foods<br>☐ Adequate water intake<br>☐ Avoiding non-recommended beverages (e.g., sodas, milk alternatives)<br>☐ Vitamin D supplementation<br>☐ Iron supplementation<br>☐ Other
Other (please specify):	
16e. What are the key nutritional messages your organization provides regarding feeding children ages 1 to 2 years? Select all that apply.	☐ Parental feeding styles<br>☐ Avoid non-recommended beverages (e.g., sodas, milk alternatives)<br>☐ Juice consumption<br>☐ Picky eating<br>☐ Adequate iron intake to reduce risk of anemia<br>☐ Healthy snacks<br>☐ Water intake<br>☐ Avoiding added sugars<br>☐ Other
Other (please specify):	
17. How are new nutrition guidelines or recommendations incorporated into the resources or services your organization provides? Select all that apply.	☐ Update education materials<br>☐ Newsletter/email to clients<br>☐ Incorporate messages into counseling sessions and opportunities<br>☐ Change in foods/supplements offered (e.g. at WIC, food banks)<br>☐ Other<br>☐ Do not know
Other (please specify):	

18. Which of the following nutrition questions or concerns do you or other providers/staff within your organization most often hear from families? Select the top 4 responses.

- ☐ Concerns of iron deficiency
- ☐ Lactose intolerance
- ☐ Breastfeeding issues
- ☐ Picky eating
- ☐ Growth and weight gain
- ☐ Reaching developmental milestones
- ☐ Supplements to use
- ☐ Excess gestational weight gain
- ☐ When to stop breastfeeding/formula
- ☐ Food allergies
- ☐ Recipes or quick meal ideas
- ☐ Other

Other (please specify):

19. Which of the following issues do you believe are most important to address in order to support the adoption and continuation of breastfeeding? select all that apply.

- ☐ Proper latching
- ☐ Nutritional needs of mom
- ☐ Reassurance that breastmilk is adequate to support healthy child growth
- ☐ Pumping / storage of breast milk
- ☐ Safe space to breastfeed or pump
- ☐ Identifying signs of hunger and satiety with baby
- ☐ Maintaining proper hydration for mom
- ☐ Access to one-to-one lactation consultations
- ☐ Other

Other (please specify):

20. Which of the following do you consider to be risk factors for breastfeeding declines or termination? Select all that apply.

- ☐ Leaving the hospital
- ☐ Baby starting daycare
- ☐ Caregiver returning to work
- ☐ Lack of home support
- ☐ Caregiver illness/depression, including postpartum
- ☐ Mother does not like pumping
- ☐ Lack of storage for pumped milk
- ☐ Other

Other (please specify):

21. Which of the following types of training and professional development opportunities does your organization offer to staff, or encourage their engagement in, around topics of supporting families' optimal nutrition? Select all that apply.

- ☐ In-house training
- ☐ Conferences
- ☐ Webinars
- ☐ College courses
- ☐ Continuing education courses
- ☐ Offsite rotations / internships
- ☐ We don't provide professional development opportunities
- ☐ Other

Other (please specify):

22. What, if any, unmet needs does your organization have in regard to providing professional development to staff?	☐ Lack of access to topic trainers<br>☐ Budgetary constraints<br>☐ Time constraints<br>☐ Understaffed<br>☐ Nutrition not prioritized in the organization<br>☐ No unmet needs<br>☐ Other
Other (please specify):	
23. Which of the following professional development topics would providers/staff within you organization benefit from on specific nutritional risks and interventions during pregnancy? Select all that apply.	☐ Adequate protein-energy intake in pregnancy<br>☐ Appropriate gestational weight gain<br>☐ Iron sufficiency during pregnancy<br>☐ Food sources that supply the critical nutrients necessary for brain development during pregnancy<br>☐ Eating a minimally processed diet<br>☐ How maternal stress affects early life nutrition and development<br>☐ Other
Other (please specify):	
24. Which of the following professional development topics would providers/staff within your organization benefit from on specific nutritional risks and interventions for 0-2 years old? Select all that apply.	☐ Appropriate growth<br>☐ At risk nutrients in breastfed infants after 6 months (i.e., zinc, iron, vitamin D)<br>☐ Food sources that supply the critical nutrients necessary for brain development<br>☐ Avoiding added sugars in the diet<br>☐ Food allergies<br>☐ Other
Other (please specify):	
25. Which California county or region do you primarily serve?	◯ Inland Empire<br>◯ Los Angeles County<br>◯ Orange County<br>◯ Sacramento<br>◯ San Diego/Imperial<br>◯ San Francisco/Bay Area<br>◯ Central Coast<br>◯ Central Valley<br>◯ Sacramento Region<br>◯ Far North<br>◯ Other
Other (please specify):	
26. Are you willing to be interviewed for the next phase of this project? All interviewees will receive a $100 check or gift card for approximately 1 hour of your time. If yes, please provide your contact information below and we will be in touch with you.	◯ Yes<br>◯ No

Thank you for your time! If you'd like to be entered into a drawing for a chance to win one of four $50 Amazon gift cards, please provide us with your contact information. We will not share this information with anyone else.

Organization Name:

Your Name:

Email Address:

Use the space to add any additional comments.
Thank You!
